# Surface-Modified Silver Nanoparticles and Their Encapsulation in Liposomes Can Treat MCF-7 Breast Cancer Cells

**DOI:** 10.3390/jfb14100509

**Published:** 2023-10-11

**Authors:** Ellenor Moors, Vinayak Sharma, Furong Tian, Bilal Javed

**Affiliations:** 1 School of Food Science and Environmental Health, College of Sciences and Health, Technological University Dublin, D07 H6K8 Dublin, Ireland; 2 Nanolab, FOCAS Research Institute, Technological University Dublin, D08 CKP1 Dublin, Ireland; 3 RELX Elsevier, D18 X6N2 Dublin, Ireland

**Keywords:** liposomes, BSA-capped AgNPs, PVP AgNPs, dipalmitoyl phosphatidylcholine lipid, cytotoxicity, MTT assay

## Abstract

Silver nanoparticles (AgNPs) have emerged as a promising tool for cancer treatment due to their unique physicochemical and biological properties. However, their clinical applications are limited by their potential cytotoxicity caused due to oxidation stress and non-specific cellular uptake pathways. To overcome these barriers, surface modifications of AgNPs have been proposed as an effective strategy to enhance their biocompatibility and specificity toward cancer cells. In this study, AgNPs were synthesised using the chemical reduction method and subsequently conjugated with various capping agents such as Polyvinylpyrrolidone (PVP) and Bovine Serum Albumin (BSA). Further, this study involves the synthesis of liposomes by using dipalmitoyl phosphatidylcholine lipid (DPPC) and cholesterol to increase the biocompatibility and bioavailability of AgNPs to MCF-7 breast cancer cells. In vitro, cytotoxicity studies were performed to determine which surface modification method exhibited the highest cytotoxic effect on the MCF-7 breast cancer cells, which was determined through the MTT assay. The AgNPs conjugated with BSA exhibited the highest cytotoxicity at the lowest dosage, with an IC_50_ of 2.5 μL/mL. The BSA-AgNPs induced a dose-dependent rise in cytotoxicity through the enhancement of nucleophilic dissolution of the AgNPs in cancer cells. In comparison, the unmodified AgNPs had an IC_50_ value of 3.0 μL/mL, while the PVP-modified AgNPs had an IC_50_ of 4.24 μL/mL. AgNPs encapsulated in liposomes had an IC_50_ value of 5.08 μL/mL, which shows that the encapsulation of AgNPs in liposomes controls their entry into cancer cells. The findings of this research have provided insights into the potential use of surface-modified AgNPs and liposomal encapsulated AgNPs as novel therapeutic tools to overcome the conventional treatment limitations of breast cancer cells.

## 1. Introduction

Breast cancer prevails when the cell in the breast grows abnormally. Breast cancer is a malignant tumour that originates in the breast tissue. Breast cancer can occur in both males and females; however, females are more at risk due to breast development and lifelong exposure to oestrogen [1]. Treatment options for breast cancer depend on the stage and type of breast cancer but typically include surgery, chemotherapy, radiation therapy, hormone therapy, and targeted therapy. Early detection through regular screenings and self-exams is crucial for the best prognosis, and treatment plans are tailored to individual patient needs [2,3].

Silver is one of the most crucial metals due to its significant role in nanotechnology, with a market value greater than USD 2 billion reported in 2020 [4]. AgNPs are well known for their antimicrobial properties and are, therefore, routinely applied in some medicines for wound dressings, medical devices, implants, the cleaning of surgical equipment, and contraceptive devices [5]. There has been an increased interest in recent years in the application of AgNPs in the targeted therapy of tumours due to their unique physical, chemical, optical, and biological properties [6]. The surface chemistry, size distribution, shape, agglomeration, and dissolution rate of nanoparticles, along with the cell type being treated, all play an important role in the biological activity of AgNPs [4]. It is crucial to create AgNPs that are homogeneous in size, morphology, and functions to improve their biological applications. However, AgNPs have the potential to exhibit cytotoxic, genotoxic, and antiproliferative effects on healthy cells along with cancerous cells [5]. Therefore, the future application of AgNPs as an antiproliferative agent in cancer treatment could be limited by the fact that it is equally toxic to normal cells. As a result, it is essential that the synthesis method of AgNPs should be controlled to generate AgNPs with homogenous size, morphology, and surface characteristics to minimise their potential adverse effects and increase their selectivity [6].

The selection of a suitable reducing agent, such as sodium citrate, ascorbate, sodium borohydride (NaBH_4_), elemental hydrogen, polyol process, and tollens reagent, is an important factor to consider, as the shape and particle size distribution of synthesised particles depends on the nature of the reducing agent [7]. Sodium borohydride (NaBH_4_) is the most commonly used reducing agent due to its low cost and availability. Sodium borohydride has been found to be effective in reducing silver ions to produce AgNPs with a size range of 5–20 nm [7]. The trisodium citrate (Na₃C₆H₅O₇) has been reported to be the most efficient reducing agent in the production of AgNPs with a size range of 60 to 100 nm [8]. However, the chemical reduction method requires the use of a metal precursor, a reducing agent, and capping agents to ensure stable chemically synthesised colloids [9].

For therapeutic use, it is necessary to utilise capping agents to control the size, agglomeration, and biological corona of the nanoparticles during colloidal synthesis and to ensure their cellular uptake and biocompatibility. The capping agents stabilise the interface between the nanoparticles and the preparation medium by creating a steric hindrance effect. Most of the capping agents are amphiphilic molecules which consist of a polar head and a non-polar hydrocarbon tail. The polar head interacts with the nanoparticle, and the non-polar tail interacts with the preparation medium [10,11]. In this study, Polyvinylpyrrolidone (PVP) was employed as a capping agent due to its ability to shield the AgNPs’ surface from protein corona formation, improving dispersion in biological media. The cellular uptake of PVP-coated AgNPs is enhanced through the control of the size and agglomeration of the particles. PVP coating prevents the aggregation of AgNPs due to the production of repulsive forces from the hydrophobic carbon chains that interact with each other, referred to as the steric hindrance effect [12]. Studies have shown that short chains of PVP produce silver nanosheets and nanoplates [13].

Another capping agent, Bovine Serum Albumin (BSA) was also used due to its stability and biocompatible nature, making it an ideal capping agent for nanoparticles. BSA has numerous binding sites due to the presence of charged functional groups, including carboxyl, sulfhydryl, and amino, which assist in the binding of nanoparticles. The binding of nanoparticles to BSA improves their bioavailability and biocompatibility within the bloodstream, which aids in their application for therapeutic use [14]. The physical adsorption of BSA onto AgNPs’ surface is through weak Van der Waal forces and hydrogen bonding. This method is simple and cost-effective and has been reported to result in stable and monodispersed particles. The BSA layer forms a protective shell around the nanoparticles, which enhances their stability, prevents aggregation, and reduces toxicity [15].

Liposomes are designed to mimic the lipid bilayer of the cell membrane, which consists of phospholipids and cholesterol molecules that help restrict the movement of small molecules. Therefore, numerous studies have encapsulated AgNPs in liposomes made of the natural biosurfactants such as dipalmitoyl phosphatidylcholine (DPPC) to increase permeabilization into the target cells, and cholesterol to enhance the rigidity of the liposomes and protect the encapsulated nanoparticles [16]. It has been reported that the prolonged administration of AgNPs could result in systemic transport to different tissues, resulting in systemic toxicity. Liposomal encapsulation improves targeted drug delivery by aiding the passive transport of nanoparticles within cells, which bypasses the cell’s membrane channels mitigating the toxic effects [5]

The main purpose of encapsulating nanoparticles in liposomes is to reduce effective doses and their associated side effects. The encapsulation of AgNPs in liposomes improves the retention effect and permeability of the vasculature [17,18]. However, the encapsulation of AgNPs alone does not ensure the specific targeting of AgNPs in cancer cells. Therefore, labelling of the surface of the liposomes with specific ligands has been developed, which targets receptors involved in the endocytosis process.

The aim of this research was to synthesise surface-modified AgNPs and liposomes to assess their relative cytotoxicity against the MCF-7 breast cancer cell line. This study identifies the potential use of surface-modified AgNPs and liposomal encapsulated AgNPs as novel therapeutic tools for breast cancer treatment. AgNPs were synthesised through the chemical reduction method using sodium borohydride, trisodium citrate, and silver nitrate. The effectiveness of various capping agents, BSA and PVP, was assessed to produce AgNPs with uniform size and morphology to minimise their cytotoxic effect. The AgNPs were encapsulated in liposomes consisting of dipalmitoyl phosphatidylcholine (DPPC) and cholesterol to enhance the permeability and rigidity of the nanoparticles within the vascular system. Findings from this study will help to improve the application potential of biocompatible surface-modified AgNPs for the treatment of breast cancer.

## 2. Materials and Methods

### 2.1. Materials and Reagents

Silver nitrate (AgNO_3_) (CAS no.: 7761-88-8), sodium borohydride (NaBH_4_) (CAS no.: 16940-66-2), trisodium citrate (CAS no.: 6132-04-3)**,** DPPC (CAS no.: 63-89-8), cholesterol (CAS no.: 57-88-5), Bovine Serum Albumin (BSA) (CAS no.: 9048-46-8), Polyvinylpyrrolidone (PVP) (CAS no.: 9003-39-8) were all purchased from Sigma-Aldrich, Dublin, Ireland. All tissue culture supplies, 96-well plates, and T75 flasks were purchased from Thermo Fisher Scientific, Dublin, Ireland. MTT (3-(4,5-dimethylthiazol-2-yl)-2,5-diphenyltetrazolium bromide) (CAS no.: 298-93-1), Trypsin (EDTA) (CAS no.: 25200072), Fetal Bovine Serum (FBS) (CAS no.: A5256701), Penicillin-Streptomycin (CAS no.: 10378016), DMEM (high glucose) (CAS no. 11965092) were purchased from Gibco, through Thermo Fisher Scientific, Dublin, Ireland.

### 2.2. Silver Nanoparticles Synthesis

AgNPs were produced via a chemical reduction of silver nitrate by sodium borohydride modified from the procedure reported by A. Yusuf et al. [5]. A 1 mM solution of AgNO_3_ and a 2 mM solution of NaBH_4_ were prepared in deionised water. The reaction was carried out in an ice bath to prevent the agglomeration of the AgNPs during the reduction process. Then, 30 mL of the 2 mM NaBH_4_ solution was added to an Erlenmeyer flask in an ice bath and stirred for 30 min. Furthermore, 6 mL solution of AgNO_3_ was added dropwise to the NaBH_4_ solution under constant stirring. After all the AgNO_3_ had been added, the flask was taken out of the ice bath and stirred until room temperature was achieved. The resulting golden yellow solution was stored at 4 °C.

### 2.3. PVP-AgNPs Synthesis

AgNPs were also capped with 0.3% PVP, which was achieved through the addition of 1 mL of 0.3% PVP solution under constant stirring for thirty minutes after the reduction reaction. The PVP-capped AgNPs were procured via centrifugation and washing of the pellet thrice to remove unbound polymer. The pellet was dissolved in the solution and stored at 4 °C until further use.

### 2.4. BSA-AgNPs Synthesis

BSA-capped AgNPs were produced by modifying the procedure reported by Dasom Kim et al. [14]. Briefly, 100 mg of BSA was dissolved in 30 mL of deionised water in a conical flask at room temperature. Then, 1 mg of NaBH_4_ was added to the conical flask as the reducing agent. Furthermore, 4 mL of 0.01 M AgNO_3_ solution was added dropwise to the BSA/NaBH_4_ solution under constant stirring. The NaBH_4_ and excess BSA were removed via centrifugation and thrice washing at 14,000 rpm for 10 min. This procedure was repeated with varying concentrations of BSA 50 mg, 25 mg, 12.5 mg, and 6.25 mg. The final BSA-AgNP samples were stored at 4 °C until further use.

### 2.5. Liposomal Preparation and Encapsulation of AgNPs

Liposomes were prepared with a combination of DPPC and cholesterol by slightly modifying the method reported by A. Yusuf et al. [5]. DPPC and cholesterol were dissolved in 5 mL of chloroform and the solution was mixed until clear. It was then dried in a vacuum oven at 52 °C overnight. The resulting lipid cake was rehydrated in 6 mL of AgNPs solution and vortexed for 10 min. The final lipid concentration obtained was 1 mg/mL of DPPC and 0.23 mg/mL of cholesterol to obtain a 7:3 molar ratio. The resulting mixture was stored at 4 °C for further use.

### 2.6. Characterisation of AgNPs

#### 2.6.1. UV-Visible Spectrophotometry

To verify the formation of AgNPs, PVP-AgNPs, Liposomal-AgNPs, and BSA-AgNPs, the UV-visible absorption spectra of the samples were detected in the range of 300–800 nm with a Perkin Elmer Lambda 900 UV-visible spectrometer. Then, 0.5 mL of the AgNPs sample and 0.5 mL of deionised water were analysed in disposable cuvettes [18].

#### 2.6.2. Particle Size Distribution and Zeta Potential Analysis

The hydrodynamic size and zeta potential of the nanoparticles were measured using Malvern Panalytical Zetasizer Nano (Worcestershire, UK). Briefly, 0.1 mL of the AgNPs’ colloidal sample was dispersed in 0.9 mL of deionised water and analysed in disposable cuvettes. For zeta potential analysis, the samples were held in capillary cuvettes. Three measurements were taken for each sample [19].

#### 2.6.3. Fluorescence Analysis

To identify the conjugation of BSA onto the NPs surface, fluorescence spectra were recorded in the wavelength range of 310–500 nm on a Perkin Elmer LS55B fluorescence spectrophotometer (USA) using an excitation wavelength of 295 nm. The excitation and emission slit widths were set at 10 nm, and the PMT voltage was set at 450 V. Then, 1 mL solution of the BSA-AgNPs sample was analysed in a quartz cuvette [9].

#### 2.6.4. Morphological Analysis Using Scanning Electron Microscopy (SEM)

The size and surface morphology of the AgNPs were examined via SEM analysis using a Hitachi SU-6600 field emission SEM. Then, 0.1 mL of the AgNPs sample was dispersed in 0.9 mL of ethanol and drop-casted on silicon wafer, which was analysed [20].

### 2.7. Cell Culture and Exposure

The MCF-7 cell line derived from human breast cancer was used for this study. MCF-7 cells were cultured in DMEM media containing L-glutamine (Sigma-Aldrich) supplemented with 10% of FBS and 1% of penicillin-streptomycin and incubated at 37 °C, with 95% humidity and 5% CO_2_ in 75 cm^3^ culture flasks. For dose-dependent treatment, the cells were seeded into a 96-well plate at a density of 1 × 10^4^ cells per well in 100 μL of culture media for 24 h. For each independent experiment, three replicate wells were performed per concentration per plate. The cells were treated with unencapsulated AgNPs, PVP-AgNPs, BSA-AgNPs, and liposome-encapsulated AgNPs after seeding. A positive control was taken, and the cells were treated by adding 25% of DMSO. A negative control of unexposed cells was also incorporated into the plate. Additional positive control was performed by exposing MCF-7 to 1–8 μL/mL of AgNO_3_ solution to test the possible cytotoxic effect of AgNPs ionisation into Ag+ after nanoparticle exposure. The plate was incubated for a further 24 h before performing the cell viability assay [21].

### 2.8. Cell Viability

After the incubation period, the culture media were removed from each well. The MTT reagent was added to serum-free DMEM to make a final concentration of 0.5 mg/mL. 100 μL of the MTT solution was added to each well. The plate was incubated for 3–4 h at 37 °C in an incubator with 95% of humidity and 5% of CO_2_ supply. After incubation, the MTT reagent solution was removed, and 100 μL of the DMSO solution was added to each well. The plate was observed for the formation of the purple formazan crystals. The resulting fluorescence of the formazan crystals was measured at 570 nm using Spectromax M3 Multi-Mode Microplate Reader and compared to the relative controls [22].

### 2.9. Statistical Analysis

All data are presented as means and ±standard errors of the mean (SEM). Statistical analysis was carried out using GraphPad Prism version 9 and Origin 8.5. Statistically significant differences in the tests were indicated for *p* < 0.05.

## 3. Results and Discussion

The primary objective was to synthesise stable AgNPs by following the conventional synthesis route. Although the green synthesis of AgNPs is more environmentally friendly due to the use of biological reagents, the chemical synthesis methods have been associated with a higher yield of AgNPs with a narrow size distribution leading to a more dispersed and homogenous population [23]. The drawback associated with the green synthesis method is the production of AgNPs with irregular surface morphologies, which affects their characterisation and surface functionalisation. The chemical reducing agent, sodium borohydride (NaBH_4_), utilised in AgNPs’ synthesis generates an even particle size distribution population and the physiochemical characteristics largely depend on the nature of the reducing agent used. Sodium borohydride was added in excess to promote instant nuclei generation and the formation of monodisperse and uniformly sized silver colloids [8].

### 3.1. UV–Visible Spectrophotometer Analysis of AgNPs and Liposomes

UV–visible analysis was performed to verify the formation of AgNPs and their modification. The surface plasmon resonance peak position and intensity are dependent on the size, shape, composition, and refractive index of the surrounding medium.

**Figure 1:**
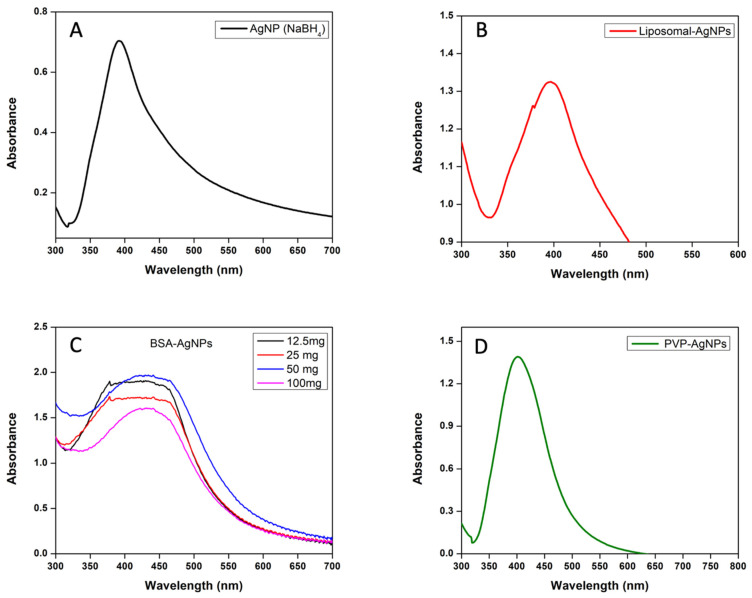
The UV–visible absorbance spectra of unmodified and surface-modified AgNPs. (**A**) AgNPs (unmodified) synthesised with NaBH_4_. (**B**) AgNPs encapsulated with liposomes. (**C**) AgNPs capped with different concentrations of BSA. (**D**) AgNPs capped with 0.3% of PVP.

The unmodified AgNPs synthesised via a sodium borohydride-mediated reduction were first analysed using UV–visible spectrophotometry to verify the formation of AgNPs. As can be seen from the UV–visible spectrum in Figure 1A, a surface plasmon resonance peak is observed at 392 nm, which is characteristic of AgNPs. The sharp peak and high intensity indicate that the size of the nanoparticles is small and monodisperse. This is because the conduction band electrons are more attracted to the crystal backbone of neighbouring AgNPs when compared to smaller particle sizes, which reduces the conduction band energy [24].

AgNPs were also encapsulated in liposomes made of DPCC lipids and cholesterol. The aim of liposomal encapsulation was to evaluate the cytotoxicity of the AgNPs through the induction of the apoptosis of cancer cells. In the case of the liposomes, the SPR peak was much broader than that of the other samples, ranging at the base from 368 nm to 469 nm (Figure 1B). This is because due to the large size of liposomes, the peak shifts to longer wavelengths (redshift) due to a decrease in the number of free electrons available for resonance. The liposomes encapsulating AgNPs have altered the size of the spherical particles, which has caused a redshift in the absorption peak to longer wavelengths.

BSA-AgNPs were synthesised to improve the stability of the synthesised AgNPs, enhance their nucleophilic dissolution and extend their plasma circulation time [25]. The concentration of BSA conjugated to the surface of the AgNPs was varied to determine whether higher concentrations of BSA had a greater impact on the prevention of agglomeration. The BSA concentration was decreased by 50% in each preparation with a starting concentration of 100 mg to determine the impact of the BSA concentration on the stability of the AgNPs. UV–visible analysis was performed to verify the formation of AgNPs capped with BSA. There is an SPR peak in all spectra between 431 and 441 nm, which is characteristic of AgNPs capped with BSA, which can be seen in Figure 1C. The presence of BSA on the surface of the AgNPs caused a redshift in the absorbance peak to longer wavelengths than those of the uncapped nanoparticles. This shift can be attributed to the interaction between BSA and the surface of the AgNPs, which alters the local refractive index and the electron density of the nanoparticles [24].

PVP stabilised the synthesised AgNPs against agglomeration after synthesis, but it also played a role in the control of the growth and nucleation of the AgNPs [25]. After the addition of PVP to the reaction mixture in the presence of sodium borohydride, the dissolution of PVP occurred rapidly, which was due to the presence of dissolved oxygen in the reaction mixture from the release of dissolved silver ions [25]. The addition of the PVP shortly after the addition of the silver nitrate prevented the further agglomeration of the AgNPs after nucleation. The SPR peak was observed at 404 nm, which can be seen in Figure 1D. The presence of PVP caused a narrow absorbance peak at a longer wavelength, which indicates the presence of unaggregated nanoparticles with a uniform size distribution. This is because the dispersed nanoparticles possess localised electrons which cause the SPR to shift to higher energies, causing the peaks to occur at longer wavelengths at higher intensities. The symmetrical absorption peak also indicates the presence of symmetrical spherical nanoparticles. The presence of the PVP on the surface of the nanoparticles has enabled the control of the surface morphology during the growth phase of the nanoparticle formation [26].

### 3.2. Fluorescence Analysis of the BSA-AgNPs

**Figure 2:**
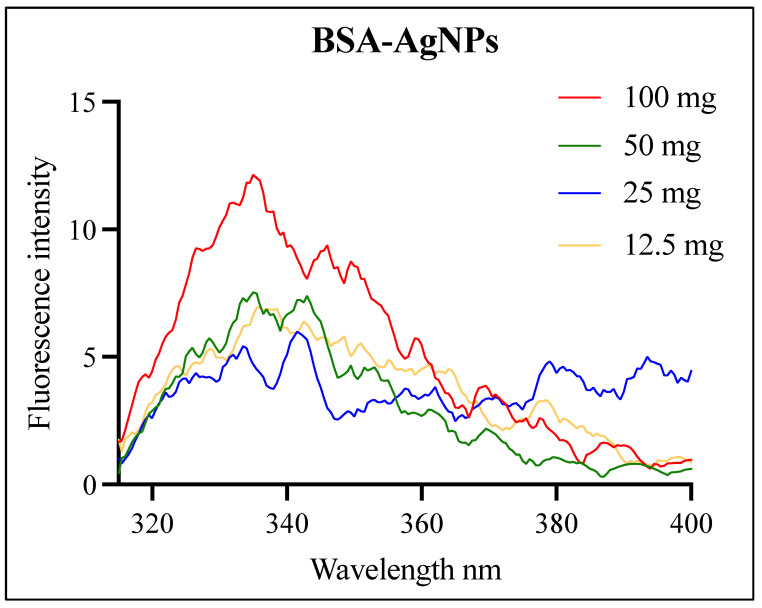
Fluorescence spectra of AgNPs capped with Bovine Serum Albumin (BSA) upon excitation at 295 nm. The concentration of BSA in each sample is diluted half-fold (100, 50, 25, and 12.5 mg).

Fluorescence analysis was performed to verify the incorporation of BSA onto the surface of the AgNPs. Figure 2 shows the fluorescence emission spectra of BSA-AgNPs upon excitation at 295 nm. It can be observed that as the concentration of BSA increases, the emission intensity increases gradually. The presence of AgNPs resulted in a decrease in the emission intensity, referred to as fluorophore quenching. Both dynamic and static quenching can be observed in these spectra. The excited state of BSA was deactivated upon contact with the AgNPs, returning it to the ground state, which is referred to as dynamic quenching. The presence of the AgNPs also forms a nonfluorescent ground state complex between the BSA and the silver, referred to as static quenching [27].

### 3.3. Particle Size Distribution Analysis and Zeta Potential of AgNPs and Liposomes

**Figure 3:**
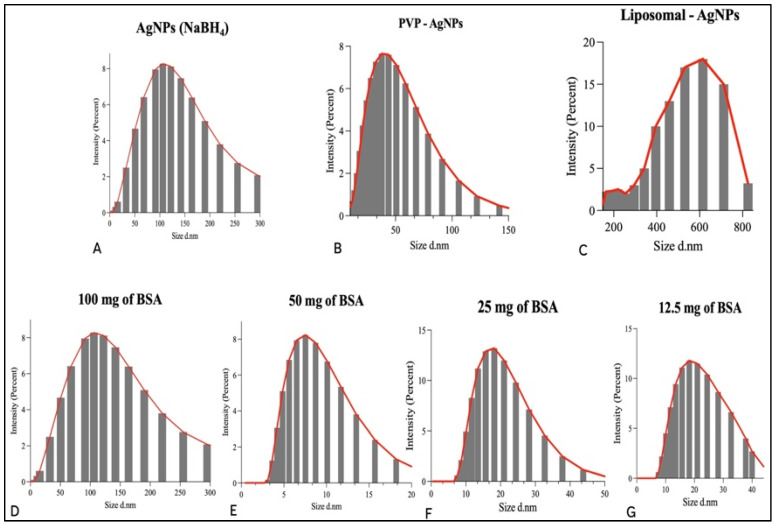
The average particle size distribution shown in intensity graphs; (**A**) average particle size distribution of AgNPs synthesised with NaBH_4_; (**B**) average particle size distribution of AgNPs capped with PVP; (**C**) average particle size distribution of AgNPs encapsulated with liposomes; (**D**–**G**) average particle size distribution of AgNPs capped with different concentrations of BSA (100 mg, 50 mg, 25 mg, and 12.5 mg).

Once the presence of AgNPs was confirmed through UV–visible analysis, DLS analysis was performed to determine the particle size, dispersity, and zeta potential of the AgNPs. The z-average size of the AgNPs was calculated to be 81 nm (Figure 3A). The size distribution of the AgNPs can be seen in Figure 3. This graph shows that there is a varied size distribution of varying intensities. The average PDI calculated was 0.37, which is an estimate of the size uniformity of the nanoparticles in the colloid solution. According to the literature, nanoparticles with a PDI value below 0.5 are acceptable for drug delivery applications [27]. The zeta potential of the AgNPs was calculated to be 41.33 mV (Table 1), which is deemed to be strongly anionic [28]. The highly negative zeta potential indicates the presence of highly charged particles, which prevents aggregation due to electrostatic repulsion. However, the highly negative charge of the nanoparticles also prevents the conjugation of biological proteins to the surface when suspended in the bloodstream. As a result, this AgNPs sample will have a shorter half-life in the bloodstream as the reticuloendothelial system can easily excrete it.

The z-average size of the PVP-capped AgNPs was calculated at 20 nm, which is significantly smaller than the unmodified nanoparticles (Figure 3B). The PDI value of 0.48 indicates that the nanoparticles are adequately uniform in size. This is due to the addition of the capping agent, which has prevented the aggregation of the particles and the subsequent formation of larger particle aggregates. The PVP has prevented the aggregation of the AgNPs due to the production of repulsive forces from the hydrophobic carbon chains that interact with each other, referred to as the steric hindrance effect [12]. The elongated chain of PVP has acted as a dispersant agent, which has prevented the agglomeration of the AgNPs [13].

The size distribution graph in Figure 3C shows a large size distribution. The calculated PDI value supports this graph with a value of 0.529. This varied size distribution is due to the varied size of the liposomes encapsulating the AgNPs. The z-average size of liposomes encapsulating AgNPs was calculated to be 1502 nm. This is significantly larger than the other AgNPs samples due to the encapsulation of the nanoparticles within the liposomes.

The z-average sizes of the AgNPs capped with different concentrations of BSA (100 mg, 50 mg, 25 mg, and 12.5 mg) were 81 nm, 17.6 nm, 18.3 nm, and 17.3, in descending order of BSA concentration. This indicates that the higher concentration of BSA has increased the adsorption ability of BSA to the surface of the AgNPs, which has increased the size diameter of the AgNPs. This hypothesis is corroborated by the size distribution graphs (Figure 3D–G). The PDI values were calculated to be 0.38, 0.45, 0.27, 0.28, in descending order of BSA concentration. The AgNPs with 25 mg of BSA have the most uniform size distribution, while the AgNPs with 50 mg of BSA have the least uniform size distribution. This indicates that a higher concentration of BSA increases the size distribution of the nanoparticles due to the variation in the BSA layer thickness around individual nanoparticles.

### 3.4. Scanning Electron Microscopic Analysis of AgNPs and Liposomes

**Figure 4:**
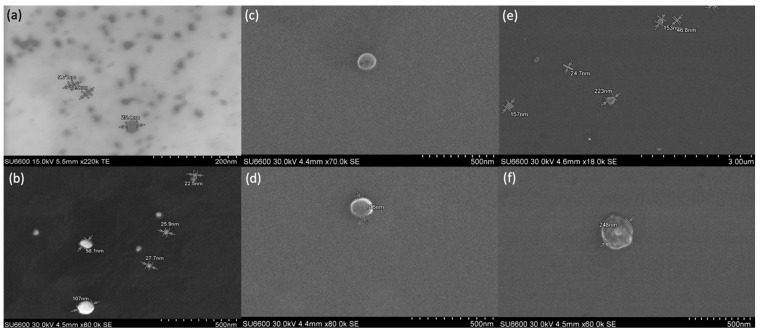
SEM images of AgNPs and liposomes; (**a**) BSA-capped AgNPs; (**b**) PVP-AgNPs; (**c**,**d**) liposomes with empty cavity; (**e**) AgNPs synthesised by using NaBH_4_; (**f**) AgNPs encapsulated in liposomes.

The SEM analysis reported silver nanostructures of different shapes and sizes. Most of the AgNPs capped with BSA and PVP appeared spherical in shape. The SEM images of AgNPs capped with 50 mg of BSA support these results. Figure 4a depicts three spherical nanoparticles of sizes 25.4 nm, 10.9 nm, and 9.71 nm. Figure 4b depicts the AgNPs’ core surrounded by a PVP corona, which indicates the successful surface modification of the AgNPs.

The SEM images of the liposomes after the addition of the AgNP sample have provided confirmation that the liposomes have encapsulated the AgNPs. Figure 4c,d showed empty liposomes. Some AgNPs of different sizes and shapes can be seen in Figure 4e. However, in Figure 4f, a dense structure can be seen inside the liposomes, which shows the successful encapsulation of nanoparticles inside the liposomes for delivery to the cancer cells.

### 3.5. Cytotoxicity Analysis of AgNPs and Liposomes

The next objective of this research was to analyse and compare the cytotoxic potential of the synthesised AgNPs and liposomes on the MCF-7 breast cancer cell line. It was determined that the optimum seeding density of the plates was 10,000 cells per well, as this density was the most cited in the literature for cytotoxicity tests on the MCF-7 cancer cell line. It was also determined that the most effective incubation period of the AgNPs treatment was 24 h. A higher seeding density was selected for a shorter period of incubation time. The reference [29] also implemented a similar treatment strategy for AgNPs cytotoxicity tests on MCF-7 cancer cells. This was therefore considered, and a 24 h treatment was implemented during the cytotoxicity analysis of the AgNP samples. Negative and positive controls were plated to ensure that there were no non-specific relations in the assay that would lead to untrue positive results. The negative control consisted of untreated MCF-7 cancer cells, which represented 100% cell viability. The positive control consisted of treatment with 25% DMSO, which represented 100% cell death. A second positive control was performed, which consisted of treatment with 100 mM silver nitrate solution (AgNO_3_). Skora et al. encapsulated AgNPs with liposomes that were labelled with epidermal growth factor to treat EGF overexpressing-cancer cells [30]. It can be seen in this study that the encapsulation of the AgNPs mitigated the toxic side effects of the AgNPs on healthy cells and enabled the specific targeting of the therapeutic agent to the cancer cells.

### 3.6. Unmodified AgNPs

**Figure 5:**
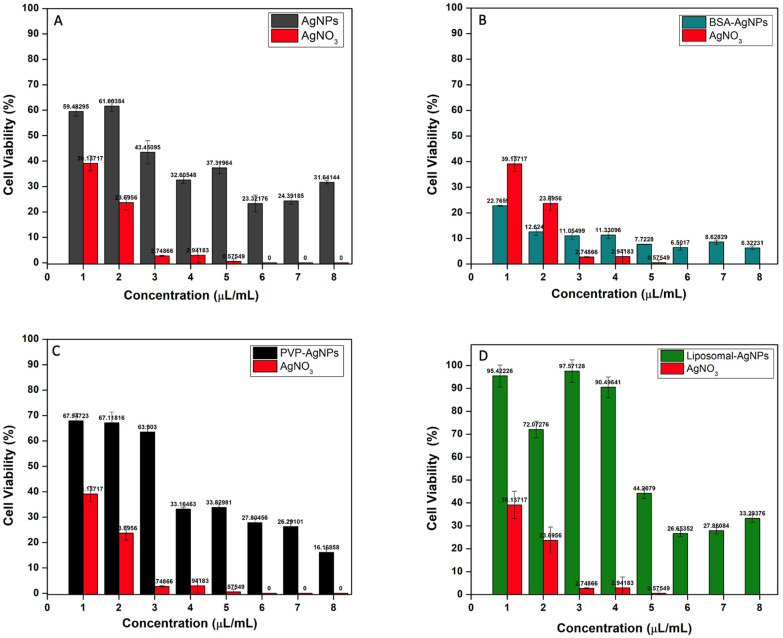
Cell viability assay in MCF-7 breast cancer cell line after 24 h of treatment. (**A**) Liposome-encapsulated AgNPs; (**B**) BSA-capped AgNPs; (**C**) PVP-capped AgNPs; (**D**) unmodified AgNPs. Note: AgNO_3_ was used as a positive control.

After exposure, the cellular viability levels of MCF-7 cells treated with varying concentrations of AgNPs for 24 h were monitored with the MTT assay. The results from the cell viability assays from two independent experiments were pooled together and displayed in Table 2. The results showed a dose-dependent reduction in cell viability with a greater level of cytotoxicity observed for the AgNO_3_-treated cells. It has been widely proposed that AgNPs’ ionisation into Ag+ is what causes the induction of cytotoxicity [16]. As a result, the cytotoxicity of Ag+ on MCF-7 cancer cells was analysed. The results show that Ag+ at a concentration of 1 μL/mL reduced MCF-7 cell viability by more than threefold. Additionally, MCF-7 cells exposed to 2 μL/mL of Ag+ showed a three-fold reduction in cell viability when compared to AgNPs (NaBH_4_) at the same concentration (Figure 5A). AgNPs and Ag+ both displayed a comparable concentration-dependent cytotoxicity profile, despite Ag+ being more hazardous. AgNPs’ slower ionisation rate, which has been shown to correlate with concentration, may be the source of its reduced cytotoxicity [31]. AgNPs have been shown to exhibit higher cytotoxicity at higher concentrations with a two-fold reduction in cell viability from 1 to 8 μL/mL of AgNPs (NaBH_4_) (Figure 5A). This is because ionisation into Ag+ is enhanced at higher concentrations [32].

### 3.7. BSA-AgNPs

**Figure 6:**
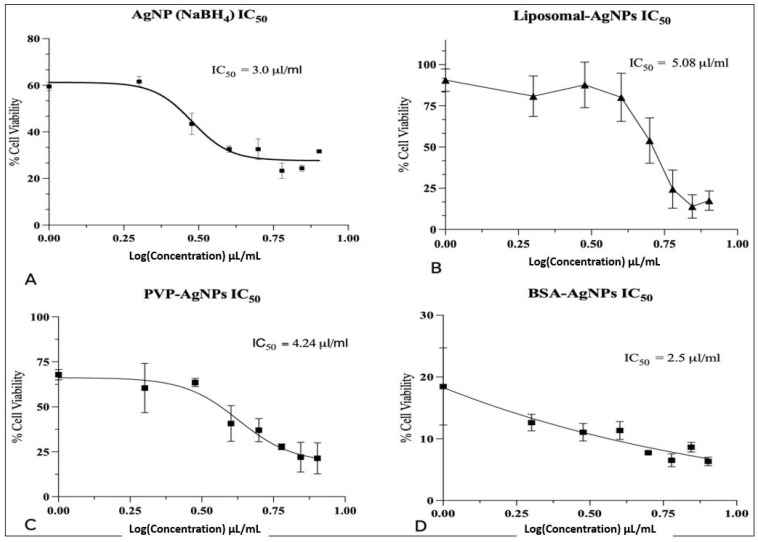
IC_50_ graphs of the cytotoxic effect produced on MCF-7 cancer cells. (**A**) IC_50_ is 3.0 µL/mL, which is the concentration required to exhibit half the max cytotoxic effect. (**B**) IC_50_ of the liposomal-AgNPs is 5.08 μL/mL. This is the largest IC_50_ value, which indicates that it produces the lowest dose-dependent rise in cytotoxicity. (**C**) IC_50_ of the PVP-AgNPs is 4.24 µL/mL. (**D**) IC_50_ of the BSA-AgNPs is 2.5 µL/mL.

The BSA-AgNPs also exhibited a greater cytotoxicity potential than the unmodified AgNPs (Figure 5B). A substantial MCF-7 cell viability reduction was observed at 1 μL/mL of the BSA-AgNP treatment, which was a three-fold reduction compared to the AgNPs (NaBH_4_) of the same concentration after 24 h. There was also a five-fold reduction in the cell viability of MCF-7 cells treated with 8 μL/mL of BSA-AgNPs compared to AgNPs (NaBH_4_) of the same concentration after 24 h, which suggests BSA-AgNPs were more cytotoxic than AgNPs at low and high concentrations. AgNPs (NaBH_4_) only induced a significant reduction in cell viability of 66.4% at 4 μL/mL after 24 h, respectively, when compared to the control. The IC_50_ values also corroborate these results, as the BSA-AgNPs have an IC_50_ of 2.5 μL/mL compared to an IC_50_ of 3.0 μL/mL for AgNPs (Figure 6).

These findings indicate that BSA-AgNPs induced a dose-dependent rise in cytotoxicity. These corroborate the findings of other studies, which suggest that BSA can enhance AgNPs’ dissolution kinetics in cancer cells in a size- and concentration-dependent manner. This is caused by an excess of BSA adhering to Ag+ and causing its dissolution at an accelerated rate. In general, BSA enhances AgNPs’ dissolution during the coating procedure. When the BSA molecules have completely coated the surface and have formed silver–sulphide linkages, further AgNP dissolution is slowed down [33]. This enhancement of nucleophilic dissolution can cause ROS generation through AgNP-catalysed free-radical reactions in cancer cells or by disturbing the biochemical equilibrium of cancer cells [34]. Therefore, it can be extrapolated that the mechanism of BSA-AgNPs’ induction of cytotoxicity in this study is caused by the formation of free radicals, which result in the production of ROS. The production of ROS causes detrimental damage to the cell plasma and mitochondrial membranes, which ultimately leads to cell death. Studies have shown that BSA can modify AgNPs dissolution kinetics in cancer cells in a size- and concentration-dependent manner. One study suggests that the oxidative release of Ag ions is enhanced by the presence of BSA on the AgNPs surface and that the degree of this enhancement is dependent on the particle size [35]. This indicates that there are size-dependent interactions occurring between biological cell components and the AgNPs surface, which lead to varying degrees of cytotoxicity [15].

The enhancement of nucleophilic dissolution can cause the generation of reactive oxygen species (ROS) through AgNP-catalysed free-radical reactions in cancer cells or by disturbing the biochemical equilibrium of cancer cells [34]. However, the generation of ROS by free AgNPs can cause adverse effects on healthy cells. As a result, the encapsulation of AgNPs within lipid bilayers has been a topic of interest to subvert the adverse effects of free AgNPs and improve targeted delivery [36]. The encapsulation of AgNPs in liposomes has been shown to increase the cytotoxicity of AgNPs at low concentrations through increased DNA damage with the suppression of ROS. This mechanism eliminates the adverse effects of ROS observed with free AgNPs [37].

### 3.8. PVP-AgNPs

The PVP-AgNPs induced a much lower level of cytotoxicity compared to the BSA-AgNPs. Evidently, there was an increase in cell viability observed for concentrations between 1 and 6 μL/mL compared to AgNPs (NaBH_4_) at the same concentrations (Figure 5C). However, at higher concentrations, 7 and 8 μL/mL, there was a greater reduction in cell viability compared to AgNPs (NaBH_4_) at the same concentrations. These results suggest that PVP-AgNPs induce a greater cytotoxic effect at higher concentrations. This is evident from the IC_50_ values, as PVP-AgNPs have an IC_50_ of 4.24 μL/mL (Figure 5C), while AgNPs (NaBH_4_) have a lower IC_50_ of 3.0 μL/mL (Figure 6). The presence of the PVP on the surface of the AgNPs may have slowed down the dissolution and ionisation of the AgNPs at low concentrations. Therefore, higher concentrations of PVP-AgNPs are required to increase the dissolution and ionisation ability. However, the administration of higher concentrations of PVP-AgNPs may result in adverse effects on healthy cells. Studies suggest that the administration of high concentrations of AgNPs results in an increase in the permeability of the mitochondrial inner membrane. Hussain et al. found that the mitochondria in rat liver treated with high concentrations of AgNPs had increased permeability, which led to swelling in the mitochondria, abnormal metabolism, and ultimately cellular apoptosis. An additional investigation discovered a large reduction in glutathione levels, a reduction in mitochondrial membrane potential, and an increase in ROS. These findings imply that oxidative stress is likely a facilitator of the cytotoxicity of AgNPs between 15 and 100 nm in liver cells [38]. Given that the PVP-AgNPs had a z-average size of 20 nm, it can be determined that the PVP-AgNPs induced cytotoxicity at high concentrations due to the production of ROS.

### 3.9. Liposomal-AgNPs

The AgNPs encapsulated in liposomes induced much less cytotoxicity than the unmodified AgNPs. As can be seen from Table 2, the liposomal-AgNPs had a 31.1% increase in cell viability compared to the unmodified AgNPs at a concentration of 1 μL/mL. However, at higher concentrations (6, 7, and 8 μL/mL), the liposomal-AgNPs had a two-fold reduction in cell viability compared to the AgNPs (NaBH_4_) at the same concentrations (Figure 5D). This suggests that the liposomal-AgNPs induce cytotoxicity at higher concentrations than the AgNPs (NaBH_4_). This is also evident from the IC_50_ values, as the liposomal-AgNPs have an IC_50_ of 5.08 μL/mL, while AgNPs (NaBH_4_) have a lower IC_50_ of 3.0 μL/mL (Figure 6). Liposomal-AgNPs’ slower ionisation rate, which has been shown to correlate with concentration, may be the source of its reduced cytotoxicity. The slower ionisation rate is due to the much larger size of the liposomal-AgNPs compared to the unmodified AgNPs. It is well known that a nanoparticle’s cytotoxic effect is influenced by its size. It was hypothesised that the uptake of nanoparticles into the cell nucleus is constrained by the nucleus’ pore size. Therefore, the size and penetrability of the liposomal-AgNPs affect their ability to induce a cytotoxic effect. Additionally, the intracellular fate of nanoparticles inside the cells is also time- and dose-dependent, in addition to being dependent on the particle size [39].

## 4. Conclusions

To summarise the findings of this research, the synthesis of AgNPs via a sodium borohydride-mediated reduction was determined to be the most effective synthesis method. This is due to the small size of the AgNPs synthesised (81 nm), their uniform dispersity (PDI 0.37), and high stability (−41.33 mZ). The AgNPs (NaBH_4_) also maintained their stability 10 weeks after synthesis. The most effective surface modification was determined to be the BSA-capped AgNPs, as these produced the highest cytotoxicity on MCF-7 breast cancer cells at the lowest concentrations with an IC_50_ of 2.5 μL/mL. The BSA-AgNPs induced a dose-dependent rise in cytotoxicity. The PVP-AgNPs and liposomal-AgNPs also exhibited a dose-dependent cytotoxic effect on the MCF-7 cancer cells, which is evident from their IC_50_ values. The low cytotoxicity of the PVP-AgNPs at low concentrations may be attributed to the presence of PVP on the surface of the AgNPs, which slowed down the dissolution and ionisation of the AgNPs at low concentrations. Therefore, higher concentrations of PVP-AgNPs are required to increase their dissolution and ionisation ability. The low cytotoxicity of liposomal-AgNPs at low concentrations may be attributed to the much larger size of the liposomal-AgNPs compared to that of the unmodified AgNPs. It is hypothesised that the uptake of liposomal-AgNPs into the MCF-7 cell nucleus was constrained by the nucleus’ pore size, which reduced its cytotoxic effect.

## Figures and Tables

**Table 1:** Average zeta potential (mV) of the unmodified AgNPs, PVP-AgNPs, and liposomal AgNPs exhibits the highest anionic charge, which indicates a higher degree of electrostatic repulsion, which prevents the aggregation of particles.

Colloidal Sample	Zeta Potential (mV)
AgNPs (NaBH_4_)	−41.33
AgNPs (NaBH_4_ and Na_3_C_6_H_5_O_7_)	−0.73
PVP-AgNPs	−33.0
Liposomal-AgNPs	−30.66
BSA 100 mg	−19.66
BSA 50 mg	18.46
BSA 25 mg	−0.07
BSA 12.5 mg	1.75

**Table 2:** Percentage viability of MCF-7 breast cancer cells against various concentrations of AgNPs.

Sample	Concentration (μL/mL)							
	1	2	3	4	5	6	7	8
AgNPs (NaBH_4_)	59.5%	63%	43%	33.6%	32.6%	23.3%	24.4%	31.5%
PVP-AgNPs	67.9%	60.4%	63.4%	40.7%	37%	27.3%	22.1%	21.4%
BSA-AgNPs	18.4%	12.6%	11.1%	11.3%	7.7%	6.4%	8.5%	5.9%
Liposomal-AgNPs	90.6%	80.5%	87%	79.5%	53.8%	24.1%	13.9%	17%
AgNO_3_	31.8%	19.8%	8.6%	2.95%	0.6%	0.38%	0.25%	0.23%

## References

[1] Han H.J., Ekweremadu C., Patel N. (2019). Advanced Drug Delivery System with Nanomaterials for Personalised Medicine to Treat Breast Cancer. J. Drug Deliv. Sci. Technol..

[2] Nounou M.I., Elamrawy F., Ahmed N., Abdelraouf K., Goda S., Syed-Sha-Qhattal H. (2015). Breast Cancer: Conventional Diagnosis and Treatment Modalities and Recent Patents and Technologies Supplementary Issue: Targeted Therapies in Breast Cancer Treatment. Breast Cancer.

[3] Lee J.H., Nan A. (2012). Combination Drug Delivery Approaches in Metastatic Breast Cancer. J. Drug Deliv..

[4] Boas D., Remennik S., Reches M. (2023). Peptide-Capped Au and Ag Nanoparticles: Detection of Heavy Metals and Photochemical Core/Shell Formation. J. Colloid Interface Sci..

[5] Yusuf A., Brophy A., Gorey B., Casey A. (2018). Liposomal Encapsulation of Silver Nanoparticles Enhances Cytotoxicity and Causes Induction of Reactive Oxygen Species-Independent Apoptosis. J. Appl. Toxicol..

[6] Sultana T., Javed B., Raja N.I., Mashwani Z.-R. (2021). Silver Nanoparticles Elicited Physiological, Biochemical, and Antioxidant Modifications in Rice Plants to Control Aspergillus Flavus. Green Process. Synth..

[7] Almatroudi A. (2020). Silver Nanoparticles: Synthesis, Characterisation and Biomedical Applications. Open Life Sci..

[8] Agnihotri S., Mukherji S., Mukherji S. (2013). Size-Controlled Silver Nanoparticles Synthesized over the Range 5–100 Nm Using the Same Protocol and Their Antibacterial Efficacy. RSC Adv..

[9] Mariam J., Dongre P.M., Kothari D.C. (2011). Study of Interaction of Silver Nanoparticles with Bovine Serum Albumin Using Fluorescence Spectroscopy. J. Fluoresc..

[10] Chen Y., Xianyu Y., Jiang X. (2017). Surface Modification of Gold Nanoparticles with Small Molecules for Biochemical Analysis. Acc. Chem. Res..

[11] Nazeer A.A., Udhayakumar S., Mani S., Dhanapal M., Vijaykumar S.D. (2018). Surface Modification of Fe_2_O_3_ and MgO Nanoparticles with Agrowastes for the Treatment of Chlorosis in Glycine Max. Nano Converg..

[12] Koczkur K.M., Mourdikoudis S., Polavarapu L., Skrabalak S.E. (2015). Polyvinylpyrrolidone (PVP) in Nanoparticle Synthesis. Dalton Trans..

[13] Tsuji M., Nishizawa Y., Matsumoto K., Kubokawa M., Miyamae N., Tsuji T. (2006). Effects of Chain Length of Polyvinylpyrrolidone for the Synthesis of Silver Nanostructures by a Microwave-Polyol Method. Mater. Lett..

[14] Kim D., Amatya R., Hwang S., Lee S., Min K.A., Shin M.C. (2021). BSA-Silver Nanoparticles: A Potential Multimodal Therapeutics for Conventional and Photothermal Treatment of Skin Cancer. Pharmaceutics.

[15] Boehmler D.J., O’Dell Z.J., Chung C., Riley K.R. (2020). Bovine Serum Albumin Enhances Silver Nanoparticle Dissolution Kinetics in a Size-and Concentration-Dependent Manner. Langmuir.

[16] Yusuf A., Casey A. (2019). Evaluation of Silver Nanoparticle Encapsulation in DPPC-Based Liposome by Different Methods for Enhanced Cytotoxicity. Int. J. Polym. Mater. Polym. Biomater..

[17] Olusanya T., Ahmad R.H., Ibegbu D.M., Smith J.R., Elkordy A.A. (2018). Liposomal Drug Delivery Systems and Anticancer Drugs. Molecules.

[18] Jiang X., Fan X., Xu W., Zhang R., Wu G. (2020). Biosynthesis of Bimetallic Au-Ag Nanoparticles Using *Escherichia Coli* and Its Biomedical Applications. ACS Biomater. Sci. Eng..

[19] Alavi M., Karimi N., Valadbeigi T. (2019). Antibacterial, Antibiofilm, Antiquorum Sensing, Antimotility, and Antioxidant Activities of Green Fabricated Ag, Cu, TiO_2_, ZnO, and Fe_3_O_4_ NPs via Protoparmeliopsis Muralis Lichen Aqueous Extract against Multi-Drug-Resistant Bacteria. ACS Biomater. Sci. Eng..

[20] Ulaeto S.B., Mathew G.M., Pancrecious J.K., Nair J.B., Rajan T.P.D., Maiti K.K., Pai B.C. (2019). Biogenic Ag Nanoparticles from Neem Extract: Their Structural Evaluation and Antimicrobial Effects against Pseudomonas Nitroreducens and Aspergillus Unguis (NII 08123). ACS Biomater. Sci. Eng..

[21] Javed B., Mashwani Z., Sarwer A., Raja N.I., Nadhman A. (2020). Synergistic Response of Physicochemical Reaction Parameters on Biogenesis of Silver Nanoparticles and Their Action against Colon Cancer and Leishmanial Cells. Artif. Cells Nanomed Biotechnol..

[22] Javed B., Mashwani Z.-R. (2020). Synergistic Effects of Physicochemical Parameters on Bio-Fabrication of Mint Silver Nanoparticles: Structural Evaluation and Action Against HCT116 Colon Cancer Cells. Int. J. Nanomed..

[23] Al-Saidi W.A., Feng H., Fichthorn K.A. (2012). Adsorption of Polyvinylpyrrolidone on Ag Surfaces: Insight into a Structure-Directing Agent. Nano Lett..

[24] Zhang X.F., Liu Z.G., Shen W., Gurunathan S. (2016). Silver Nanoparticles: Synthesis, Characterization, Properties, Applications, and Therapeutic Approaches. Int. J. Mol. Sci..

[25] Caraceni P., Tufoni M., Bonavita M.B. (2013). Clinical Use of Albumin. Blood Transfus..

[26] Loza K., Diendorf J., Sengstock C., Ruiz-Gonzalez L., Gonzalez-Calbet J.M., Vallet-Regi M., Köller M., Epple M. (2014). The Dissolution and Biological Effects of Silver Nanoparticles in Biological Media. J. Mater. Chem. B.

[27] Danaei M., Dehghankhold M., Ataei S., Hasanzadeh Davarani F., Javanmard R., Dokhani A., Khorasani S., Mozafari M.R. (2018). Impact of Particle Size and Polydispersity Index on the Clinical Applications of Lipidic Nanocarrier Systems. Pharmaceutics.

[28] Clogston J.D., Patri A.K. (2011). Zeta Potential Measurement. Methods Mol. Biol..

[29] M J.F., P L. (2015). Apoptotic Efficacy of Biogenic Silver Nanoparticles on Human Breast Cancer MCF-7 Cell Lines. Prog. Biomater..

[30] Skóra B., Piechowiak T., Szychowski K.A. (2022). Epidermal Growth Factor-Labeled Liposomes as a Way to Target the Toxicity of Silver Nanoparticles into EGFR-Overexpressing Cancer Cells in Vitro. Toxicol. Appl. Pharmacol..

[31] Maurer-Jones M.A., Mousavi M.P.S., Chen L.D., Bühlmann P., Haynes C.L. (2013). Characterization of Silver Ion Dissolution from Silver Nanoparticles Using Fluorous-Phase Ion-Selective Electrodes and Assessment of Resultant Toxicity to Shewanella Oneidensis. Chem. Sci..

[32] De Matteis V., Malvindi M.A., Galeone A., Brunetti V., De Luca E., Kote S., Kshirsagar P., Sabella S., Bardi G., Pompa P.P. (2015). Negligible Particle-Specific Toxicity Mechanism of Silver Nanoparticles: The Role of Ag+ Ion Release in the Cytosol. Nanomedicine.

[33] Liu C., Leng W., Vikesland P.J. (2018). Controlled Evaluation of the Impacts of Surface Coatings on Silver Nanoparticle Dissolution Rates. Environ. Sci. Technol..

[34] Nogueira D.R., Mitjans M., Rolim C.M.B., Vinardell M.P. (2014). Mechanisms Underlying Cytotoxicity Induced by Engineered Nanomaterials: A Review of In Vitro Studies. Nanomaterials.

[35] Moon J., Kim G., Lee S. (2012). A Gold Nanoparticle and Aflatoxin B1-BSA Conjugates Based Lateral Flow Assay Method for the Analysis of Aflatoxin B1. Materials.

[36] Javed B., Raja N.I., Nadhman A., Mashwani Z.-R. (2020). Understanding the Potential of Bio-Fabricated Non-Oxidative Silver Nanoparticles to Eradicate Leishmania and Plant Bacterial Pathogens. Appl. Nanosci..

[37] Javed B., Nadhman A., Razzaq A., Mashwani Z. (2020). One-Pot Phytosynthesis of Nano-Silver from *Mentha longifolia* L.: Their Characterization and Evaluation of Photodynamic Potential. Mater. Res. Express.

[38] Hussain S.M., Hess K.L., Gearhart J.M., Geiss K.T., Schlager J.J. (2005). In Vitro Toxicity of Nanoparticles in BRL 3A Rat Liver Cells. Toxicol. Vitr..

[39] Marcelo G.A., Lodeiro C., Capelo J.L., Lorenzo J., Oliveira E. (2020). Magnetic, Fluorescent and Hybrid Nanoparticles: From Synthesis to Application in Biosystems. Mater. Sci. Eng. C.

